# Design, synthesis and screening of indole acetic acid-based tri-azo moieties as antioxidants, anti-microbial and cytotoxic agents

**DOI:** 10.3389/fphar.2023.1084181

**Published:** 2023-02-27

**Authors:** Maryam Javaid, Ihsan-Ul Haq, Humaira Nadeem, Humaira Fatima, Arif-Ullah Khan, Nadeem Irshad

**Affiliations:** ^1^ Department of Pharmacy, Faculty of Biological Sciences, Quaid-i-Azam University, Islamabad, Pakistan; ^2^ Riphah Institute of Pharmaceutical Sciences, Riphah International University, Islamabad, Pakistan

**Keywords:** 1, 2, 4-triazoles, Schiff base, antioxidant, antibacterial, cytotoxic. protein kinase inhibition

## Abstract

Multidrug resistance and infectious disease have enormous spread despite drug discovery and development advancements. 1, 2, 4 -triazoles have been extensively studied, playing an imperative role in many pathologic conditions. A series of Schiff base triazoles; derived from Indole -3- acetic acid with substituted Benzaldehydes (5a-5g) were designed, synthesized, and evaluated through various Spectroanalytical techniques. SwissADME was used to assess physicochemical properties and pharmacokinetic drug-likeliness behavior. (5a-5g) were evaluated for their varied biological potential through antioxidant, antimicrobial, enzyme inhibition, and cytotoxic evaluation. Schiff bases express drug-like nature as they follow Lipinski’s rule of five. **5b** showed good antioxidant potential in total antioxidant capacity (TAC) and total reducing power (TRP) assays and was most active in the library in % free radical scavenging assay (%FRSA), showing 32% inhibition at 50 μg/mL concentration. Compounds showed antibacterial activity against various tested strains. **5e and 5f** showed a minimum inhibitory concentration (MIC) value of 3.12 μg/mL for *P.aeruginosa* and *K*.*pneumoniae*, respectively. In the antifungal assay, only **5e** inhibited one strain with a zone of inhibition >6 mm. These synthetic molecules possess good cytotoxic potential in the Brine Shrimp Lethality screening; **5c, 5d, and 5f** exhibited LC_50 =_5.7 μg/mL. In the protein kinase inhibition assay, **5a, 5b,** and **5g** demonstrated inhibitory potential, showcasing the zone of inhibition as 7.5–10.5 mm for the bald one and 6–7.5 for the clear zone. These findings suggest that the compounds have antibacterial and cytotoxic potential, and there is a chance for further research and development in this area.

## 1 Introduction

Developing new strategies and advancements in combinatorial chemistry has led to substantial progress in the control and treatment of microbial infections. However, microbial resistance is still a significant concern for scientific communities and threatens public health (Wang et al., 2022). Resistant pathogens are a potential threat to increased morbidity and mortality. With increased multidrug resistance, this scenario has led to an increased urge to synthesize new drugs with improved safety profiles. To combat multidrug resistance, the dire need is to develop new classes of anti-microbial agents with unique mechanisms of action (Idhayadhulla et al., 2014). Medicinal chemists are dealing with different approaches to counter this issue, including preying on new targets, structural adaptations, and joining different pharmacophores to create hybrid molecules, the most effective strategy being linkage through covalent bonds (Alkhzem et al., 2022). An imbalance between oxidants and antioxidants in our body is called “Oxidative stress,” which, in some cases, may affect essential tissue proteins and genomic matter (Lobo et al., 2010). Reactive Oxygen or Nitrogen species may be formed in the body due to certain pathophysiological conditions, causing damage to cell structures and leading to unwanted cell growth and division (Mistry et al., 2017). As a result, free radicals are produced, which can act as oxidants; antioxidants play their role, douse these oxidants, and show their significance in opposing carcinogenesis (Kattappagari et al., 2015).

Heterocyclic compounds constitute a significant part of organic chemistry. A primary concern of modern scientists is the development of new classes of heterocycles keeping environmental and financial issues in view. Heterocycles containing three hetero atoms at symmetrical positions are more widely studied because they show various pharmacological activities (Deodware et al., 2021). Nitrogen-containing heterocycles are gaining much attention as part of many approved drugs; triazoles have shown various therapeutic activities. Triazole is a 5-membered ring structure that contains three nitrogen and two Carbon atoms, and may exist as two isomers; 1, 2, 4-triazole and 12, 3-triazole. Out of these1, 2, 4-triazoles are widely investigated and reported because of their significant binding potential and stability (Strzelecka and Swiatek, 2021). 1, 2, 4-triazole containing heterocycles and those which possess 1, 2, 4-triazoles as condensation products with another nucleus system form a very diverse class of compounds, which possess antibacterial, antifungal, anti-inflammatory, and antitumor activities. Moreover, they have been reported as CNS depressants, antiproliferative, anti-HIV, anti-tubercular, analgesic, antioxidant, and anti-inflammatory (Matin et al., 2022). Several other drugs have been reported consisting of 1, 2, 4 Triazole nucleus; these include anti-psychotic, anti-migraine, sedative and hypnotic, anti-depressant, antiviral, analgesic, and aromatase inhibitors (Aggarwal and Sumran, 2020).Schiff bases are documented with an inclusive range of chemotherapeutic doings; azomethine on Schiff bases attributes to their extended chemical and biological properties Moreover, the ability to form intermolecular hydrogen bonds and transferable protons play their part in extended bioactivity (Said et al., 2021).Schiff bases are derived from aliphatic and aromatic aldehydes, but later are more stable due to conjugation, and the former are liable to polymerization (Hasan et al., 2015).Heterocyclic Schiff bases have been reported as: antibacterial, anti-proliferative (Al-Hiyari et al., 2021), antioxidant (Kizilkaya et al., 2020) antifungal (Shafiei et al., 2021) antiviral (Alotaibi et al., 2022), anti-inflammatory (Hamid and Salih, 2022) and anti-tumoral (Iacopetta et al., 2021).

**FIGURE 1:**
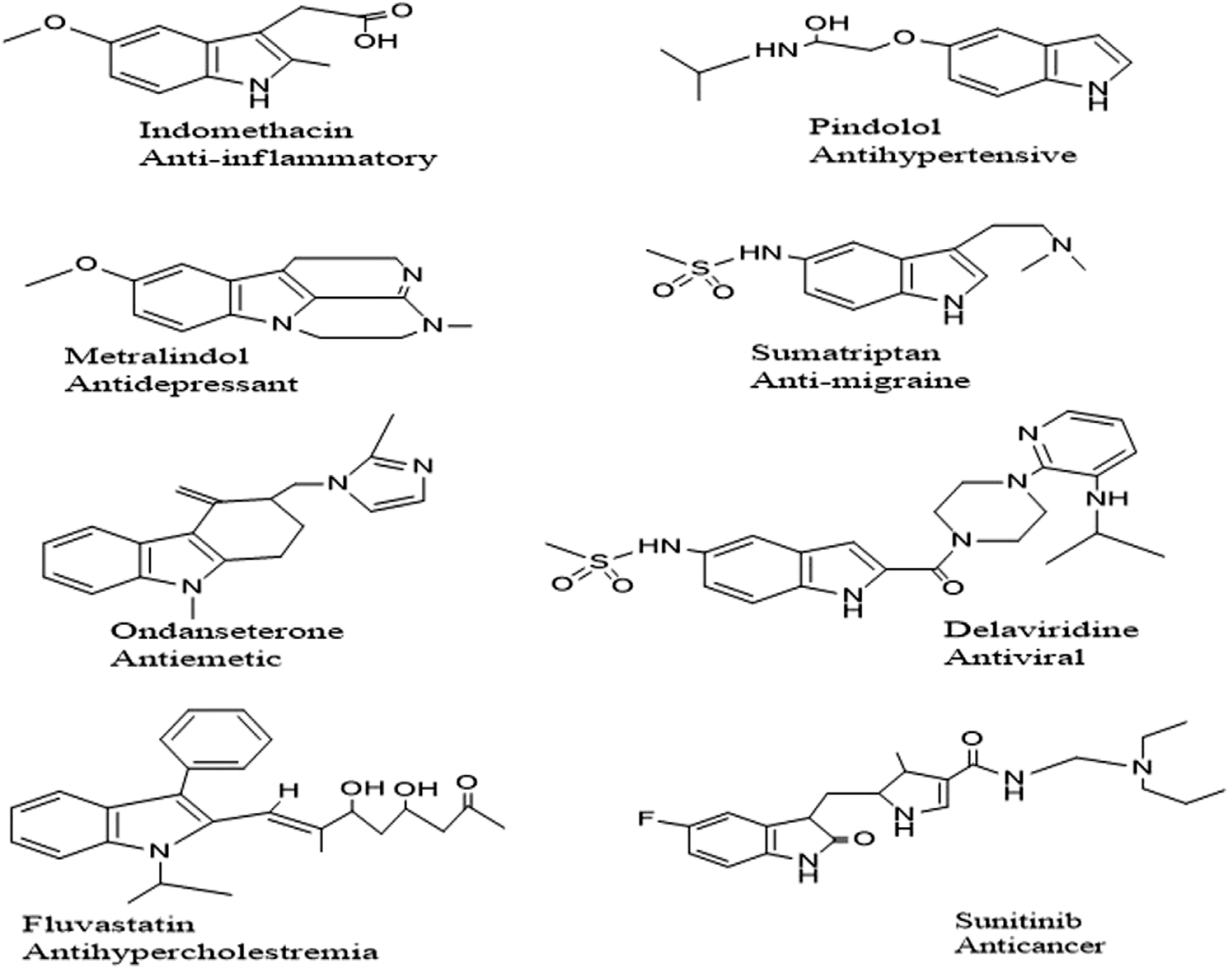
Selected Examples of FDA approved Indole based drugs.

Indole is one of the most common naturally occurring Nitrogen moieties known for its diverse biological and chemical facets. Many pharmacological activities have been reported: antibacterial, antifungal, antioxidant, antidiabetic (Zhu et al., 2021), anticancer (Yousif et al., 2019) antitubercular (Ramesh et al., 2020) ulcerogenic and anti-inflammatory (Bhat et al., 2020). Indole nucleus is associated with polypharmacological activities; a few commercially available drugs are enlisted in Figure 1. Compounds incorporating the S atoms in their heterocyclic ring system have been presented as strong candidates for medicinal chemistry endeavors because of their diverse biological potential (Boraei et al., 2020). Sulfur-containing compounds are part of many natural and synthesized drug molecules.

Influenced by these observations and compounds containing nitrogen, sulfur, and heterocyclic ring structures have shown promising therapeutic activities, this study intends to synthesize new compounds by combining moieties with pharmacological compatibilities and chemical veracity. Indole -3- acetic acid was used as starting material, and after its conversion into respective 1, 2, 4, triazole, Schiff bases were prepared by treatment with various benzaldehydes. The resulting compounds were recrystallized and characterized using Fourier Transform Infrared spectroscopy (FTIR), Proton and Carbon Nuclear Magnetic Resonance Spectroscopy (^1^H-NMR), (^13^C-NMR), and elemental analysis. These compounds were further screened for biological potential as antibacterial, antifungal, protein kinase inhibitors, and antioxidants, and for their cytotoxic potential.

## 2 Materials and methods

### 2.1 Chemistry

Commercially available chemicals were procured from Sigma Aldrich and used without refinement. M.P. was determined in sealed capillaries on a digital Gallen Kamp SAYO model MPD BM 3.5 apparatus and are uncorrected; I.R. spectra were recorded at alpha Bruker FT-IR spectrophotometer (ATR eco ZnSe, Vmax in cm ^-1^, (Germany), ^1^HNMR and ^13^C NMR spectra were elucidated *via* Bruker AV_400 spectrophotometer in DMSO at 400 MHz using tetramethyl silane (TMS); an internal standard. COSTECH Elemental Combustion System CHNS-O Elementalanalyzer from PINSTECH was used for Combustion analysis. Compound purity was assessed through thin-layer chromatography

#### 2.1.1 2- (1H-indol-3-yl) acetohydrazide

Indole -3- acetic acid (10.0 g) and 85% hydrazine hydrate (10.0 g) as a mixture was refluxed for 12 h at 80°C. Progress of reaction and completion was observed with the TLC system (Ethyl acetate: n-hexane: 1:1), the resultant precipitate was filtered, and the filtrate was washed with n-Hexane to give compound P_1_.

Yield 80%, MP 116°C, ^1^HNMR (DMSO -d6, 400 MHz) 10.1 (s, 1H, indole-NH), 8.00 (s, 1H, Indole-sec amine) 7.32–7.18 (m, 2H, Ph-H) 3.73 (s, 2H, -CH_2_-) ^13^C NMR (DMSO-d6, 400 MHz) 124.1, 135.5, 102.1, 127.6, 111.0, 120.5, 119.6, 121.7, 165.0, 35.9 IR t_max_ 3051, 3260, 3387, 1620, 1541, 1378 cm ^-1^ Elemental analysis (calculated) for C_10_H_11_N_3_O C 63.43, H 5.87, N 22.221, O 8.46, M.S. Est m/z ratio 189.0

#### 2.1.2 5-((1H-indol-3-yl) methyl)-1, 3, 4-oxadiazole-2-thiol

A mixture of P1 (10 g) and carbon disulfide 3.1 g in 95% ethanol was refluxed for 13 h to maintain a basic media, KOH (3 g) was added. Once a single spot was obtained on TLC (3:1, n-Hexane: Ethyl acetate), the temperature of the reaction mix. was lowered to room temperature, and upon treatment with conc. HCl pH was maintained. The contents were filtered, and washed with methanol. The result was P2.

Yield 79%, M.P. 125 C,^1^HNMR (DMSO-d6,400 MHz) 13.05 (s, 1H, S.H.) 10.1 (s, 1H, Indole -N.H.), 7.60–7.11 (m, 4H, Ph-H), 3.69 (s, 2H, -CH_2_-), ^13^CNMR 166.4, 124.1,135.5,102.1,127.6,111,120.5,119.6, 121.7, I.R. t_max_ 3395, 3198,2183,1617, 1541,1275,1217, 1092, Elemental analysis calc for C_11_H_9_N_3_OS C 57.12; H 3.93; N 18.14; O 6.92; S 13.89, M.S. Est m/z ratio 231.05.

#### 2.1.3 5-((1H-indol3-yl) methyl)-4-amino-4H-1, 2, 4-triazole-3-thione

A mixture of P2 and 85% hydrazine Hydrate (1:1) in ethanol was refluxed for 12 h; reaction development was supervised through TLC using Ethyl acetate and n-Hexane. The resultant precipitates were filtered off and washed with n-Hexane.

Yield 85%, M.P. 175°C, ^1^HNMR (DMSO-d6,400 MHz)13.79 (s, 1H, -S.H.), 10.1 (s, 1H, indole-NH) 5.77 (s, 2H, indole amine) 7.60–7.11 (m, 4H, Ph-H), 3.89 (s, 2H, -CH_2_-) ^13^CNMR; 166.8,152.0,123.0,136.5, 108.1, 127.4, 111.1, 118.8, 121.7, 119.8, 22.1, IR_tmax_ 3395, 3198, 3031, 1629, 1541, 1452_,_ 1277, Elemental analysis calc for C_11_H_11_N_5_S, C,53.83; H,4.55; N, 28.54; S, 13.07; M.S. Est m/z ratio 245.07.

#### 2.1.4 General synthetic procedure for Schiff Bases (5a-5g)

A solution of corresponding compound P3 and appropriate aldehyde in ethanolic media was refluxed for 8–10 h; glacial acetic acid was used as a catalyst. After cooling the mixture to room temperature and removing the solvent under reduced pressure, the crude product was recrystallized from Ethyl acetate. TLC was used to monitor the reaction progress using n-Hexane and Ethyl acetate (1:2) as mobile phase.

##### 2.1.4.1 3-((1 H-indol-3-yl) methyl)-4-((3- hydroxybenzylidene) amin o-1 H-1, 2, 4-triazole-5 (4H)-thione (5a)

% Yield 74%, M.P. 215°C, ^1^HNMR (DMSO-d6,400 MHz) 3.579 (s, 1H,-CH2),9.906 (s, 1H, = C.H.), 6.99–7.88 (m, Aryl H =8H), 10.852 (s, 1H, -N.H.) ^13^CNMR 193.23, 173.43, 158.14, 157.78, I.R. t_max_ 3207 (-N.H.), 1657 (-C=N), 1276 (-C=S), Elemental analysis calc for C_18_H_15_N_5_OS C, 61.86; H, 4.35; N, 20.08; O,4.53; S, 9.18 Found: C, 61.89, H,4.32; N, 20.12; O; 4.57; M.S. Est m/z 49.10 (100.0%), 350.10 (22.2%), 351.11(1.8%)

##### 2.1.4.2 3-((1 H-indol-3-yl) methyl)-4-((3- methoxybenzylidene) amino-1 H-1, 2, 4-triazole-5 (4H)-thione (5b)

% Yield 72%, M.P. 213°C, ^1^HNMR (DMSO-d6,400 MHz) 3.579 (s, 1H,-CH2), 9.906 (s, 1H, = C.H.),6.99–7.88 (m, Aryl H =8H), 10.852 (s, 1H, -N.H.) ^13^CNMR 193.23, 173.43, 158.14, 157.78, I.R. t_max_ 3207 (-N.H.), 657 (-C=N), 1276 (-C=S), Est m/z: 349.10 (100.0%), 350.10 (22.2%), 351.10 (5.3%), 351.11 (1.8%); Elemental Analysis for C_18_H_15_N_5_OS: C, 61.89; H, 4.34; N, 20.08; O, 4.56; S, 9.18

##### 2.1.4.3 3-((1 H-indol-3-yl) methyl)-4-((4-chlorobenzylidene) amino-1 H-1, 2, 4-triazole-5 (4H)-thione (5c)

% Yield 75%, M.P. 219°C, ^1^HNMR (DMSO-d6,400 MHz) 3.579 (s, 1H,-CH2), 9.906 (s, 1H, = C.H.), 6.99–7.88 (m, Aryl H =8H), 10.852 (s, 1H, -N.H.) ^13^CNMR 193.23, 173.43, 158.14, 157.78, I.R. t_max_ 3207 (-N.H.), 657 (-C=N), 1276 (-C=S),m/z: 367.07 (100.0%), 369.06 (36.5%), 368.07 (20.4%), 370.07 (6.3%), 369.07 (2.3%), 368.06 (1.8%), 370.06 (1.8%), 371.06 (1.6%); Elemental Analysis for C18H15ClN5S: C, 58.72; H, 3.83; Cl, 9.62; N, 19.07; S, 8.79

##### 2.1.4.4 3-((1 H-indol-3-yl) methyl)-4-((4-nitrorobenzylidene) amino-1 H-1, 2, 4-triazole-5 (4H)-thione (5d)

% Yield 75%, M.P. 220°C, ^1^HNMR (DMSO-d6,400 MHz) 3.579 (s, 1H,-CH2), 9.906 (s, 1H, = C.H.), 6.99–7.88 (m, Aryl H =8H), 10.852 (s, 1H, -N.H.) ^13^CNMR 193.23, 173.43, 158.14, 157.78, I.R. t_max_ 3207 (-NH) 657 (-C=N), 1276 (-C=S), Elemental analysis for C_18_H_14_N_6_O_2_S; C 57.18; H, 3.77; N, 22.29; O, 8.46; S,8.47; M.S. Est m/z: 378.09(100.0%), 379.09(22.6%), 380.11(5.5%), 380.11(1.8%).

##### 2.1.4.5 3-((1 H-indol-3-yl) methyl)-4-((4- hydroxybenzylidene) amino-1 H-1, 2, 4-triazole-5 (4H)-thione (5e)

% Yield 77%, M.P. 215°C, ^1^HNMR (DMSO-d6,400 MHz) 3.693 (s, 1H, -CH2), 9.881 (s, 1H, = C.H.), 7.1–7.81 (m, Aryl H =8H), 10.863 (s, 1H, -NH) ^13^ CNMR 193.77, 173.80 158.30, 138.03, I.R. t_max_ 3207 (-N.H.), 1657 (-C=N), 1276 (-C=S), Elemental analysis calc for C_18_H_15_N_5_OS C, 61.85; H, 4.36; N, 20.09; O,4.53; S, 9.15; M.S. Est m/z 349.10 (100.0%), 350.10 (22.2%), 351.11(1.8%)

##### 2.1.4.6 3-((1 H-indol-3-yl) methyl)-4-((3-chlorobenzylidene) amino-1 H-1, 2, 4-triazole-5 (4H)-thione (5f)

% Yield 75%, M. P 219°C, ^1^HNMR (DMSO-d6,400 MHz) 3.693 (s, 1H, -CH2), 9.881 (s, 1H, = C.H.), 7.1–7.81 (m, Aryl H =8H), 10.863 (s, 1H, -N.H.),^13^CNMR 193.77, 173.80 158.30, 138.03, I.R. t_max_3207 (-N.H.), 1657 (-C=N), 1276 (-C=S), M.S. Est m/z: 349.10 (100.0%), 350.10 (22.2%), 351.11(1.8%)

##### 2.1.4.7 3-((1 H-indol-3-yl) methyl)-4-((3,4,5-trimethoxybenzylidene) amino-1 H-1, 2, 4-triazole-5 (4H)-thione (5g)

% Yield 71%, M.P. 220°C, ^1^HNMR (DMSO-d6,400 MHz) 3.693 (s, 1H, -CH2), 9.881 (s, 1H, = C.H.), 7.1–7.81 (m, Aryl H =8H), 10.863 (s, 1H, -N.H.) ^13^CNMR 193.77, 173.80 158.30, 138.03, I.R. t_max_ 3207 (-N.H.), 1657 (-C=N), 1276 (-C=S), Est m/z: 409.12 (100.0%), 410.12 (24.3%), 411.12 (5.1%), 411.13 (2.9%), 412.12 (1.1%; Elemental Analysis for C_20_H_19_N_5_O_3_S: C, 58.63; H, 4.64; N, 17.13; O, 11.77; S, 7.87

### 2.2 In silico studies

Using an available software (SWISS ADME), pharmacokinetic parameters were determined. Employing a computer-aided program, we assessed the newly synthesized compounds for five different parameters: Molecular Weight (Mol.wt.). *Moriguchi* log of the Partition coefficient (MLOGP), octanal-water partition coefficient. (AlogP), hydrogen bond acceptor (H-BA) and donor (H-BD) and drug likeliness according to Lipinski’s rule of five (RO5).

### 2.3 Pharmacological evaluation

#### 2.3.1 Antioxidant estimation

##### 2.3.1.1 DPPH assay

The DPPH assay was executed *via* the technique stated by (Bibi et al., 2011). DPPH(Diphenyl-1-picrylhydrazyl), a free radical, is bagged to inquire about the antioxidant ability of the synthesized compounds. 10 μL from the synthesized compound (1 mg/mL DMSO) is added to 190 μL DPPH solution., making up the final conc. of 50 μg/mL. Absorbance was observed at 517 nm using a microplate reader after incubating at 37 C for 30 min approx. the given formula was computed

%Free radical Scavenging Activity=1−As/Ac * 100

DPPH assay was repeated in triplicate using DMSO as -ve and ascorbic acid as + ve control; findings are expressed as means standard deviation.

##### 2.3.1.2 Assessment of total antioxidant capacity (TAC assay)

This evaluation was based on the protocol by (Bibi et al., 2011). This mechanism’s key point is reducing Mo (VI) to Mo (V). A solution consisting of 4 mM Ammonium molybdate, 28 mM SodiumPhosphate, and 0.6 M Sulphuric acid is utilized, which forms Phosphate Molybdenum Complex which is green in color and gives absorption = 630 nm. 20 mL of the sample is added to 180 mL of reagent, which is incubated at 95°C for one and a half hours and cooled to 25°C.

##### 2.3.1.3 Approximation of total reducing power (TRP assay)

The process used for TRP was illustrated by (Bibi et al., 2011). The potassium ferrocyanide calorimetric method is used for 100 μL of each solution, 200 μL Phosphate buffer (0.2 M, pH 6.6), and 250 μL of potassium ferricyanide was mixed and incubated at 50°C for 20 min, to this solution, 10 percent (200 μL) trichloroacetic acid was added, centrifugation was done at 3,000 rpm for 10 min, the supernatant was collected, added with FeCl_3_ (50 μL), and shifted to the microplate. Absorbance was recorded at 700 nm. Ascorbic acid (100 μg/mL DMSO) is the +ve control for the test, and dimethyl sulfoxide (DMSO) is the blank.

#### 2.3.2.Antimicrobial assay

##### 2.3.2.1 Antibacterial assay

The micro broth dilution method was used to establish the antibacterial potential of test samples against *Staphylococcus aureus* (ATCC-6538), *Bacillus subtilis* (ATCC-6633), *Escherichia coli* (ATCC-25922), *Klebsiella pneumoniae* (ATCC-1705)*,* and *Pseudomonas aeruginosa* (ATCC-15442) and for the resistant strains of *MRSA Staphylococcus aureus, P. aeruginosa* and *Escherichia coli*. The procedure used is described by (Sarker et al., 2007). Bacterial inoculum was formed under aseptic conditions keeping the density near 5 × 10^4^ CFU/mL in pre-autoclaved nutrient broth. Test samples of an aliquot of 5 µL were shifted to labeled wells of 96 well plates, and after that addition of 195 µL of nutrient broth; was accomplished. After that, a 2-fold serial dilution of every sample was arranged to get final concentrations of 12.5 μg/mL. Then, with the help of a micropipette, 195 µL of bacterial culture of respective strains was poured into each well; further advancement was incubating of plates for 30 min at the temperature of 37°C. Zero-time readings at 630 nm were observed with the help of a microplate reader. Incubation of plates was again carried out at 37°C for 1 day, absorbance was noted, and net change in turbidity was the difference between two values of absorbance.

##### 2.3.2.2 Antifungal examination

The antifungal response to these chemical moieties was evaluated through a procedure designated by (Zahra et al., 2017). Amphotericin B (4 mg/mL DMSO) is used as + ve control and DMSO as-ve one (5 μL/disc). Sterile SDA-coated plates were used, on which 100 µL of suspension of spores for each strain (previously reaped-in-Tween-20 solution) was spread. Discs of sterile filter paper containing 5 µL of sample solution (4 mg/mL DMSO) were kept on plates and incubated for 2 days at 28°C. Zones of inhibition were recorded.

#### 2.3.3 Investigation of the compounds for cytotoxic attributes

##### 2.3.3.1 Brine Shrimp Lethality assessment

The cytotoxic evaluation was accomplished using the decorum designed by (Khan et al., 2011). To a plate of 96 wells, artificial seawater and nauplii were added. Test samples were taken in wells (40, 20, 10, 5 μg/mL) after making up the final volume with seawater, the plates were left uncovered under the lamp, and after 24 h, the number of surviving larvae was calculated. For samples having ≥50% mortality, the median lethal conc was calculated using the table curve software, Doxorubicin was used as + ve control, and DMSO was the -ve. The percentage of killed organisms was found using the formula.

Percentage Mortality=No of dead shrimps÷Total Number of Shrimps×100

##### 2.3.3.2 Protein kinase inhibition assay

The protocol followed for this is mentioned (Fatima et al., 2015). To assess the test compound’s inhibitory potential, *Streptomyces* 85 E strains were used, which were refreshed in tryptone soya broth at 37 for 1 day. Spores from refreshed cultures were utilized to prepare the bacterial culture. 100 μg compound was loaded on 5 mm discs of filter paper. These were kept on sowed plates. DMSO-permeated discs were the -ve control. The incubation period was 7 days, and outcomes were stated as bald and clear zones of inhibition.

### 2.4 Statistical analysis

The results of all the experiments were articulated as mean ± SD. Each test was performed in triplicate. TAC assay was analyzed by one-way analysis of variance (ANOVA) following DUNCAN’S test using the statistical package for social sciences 18 and *p* < 0.05 was considered as significant where appropriate. For samples having ≥50% mortality, the median lethal concentration (LC_50)_ was calculated using the table curve software.

## 3 Result and discussion

### 3.1 Chemistry

**FIGURE 2:**
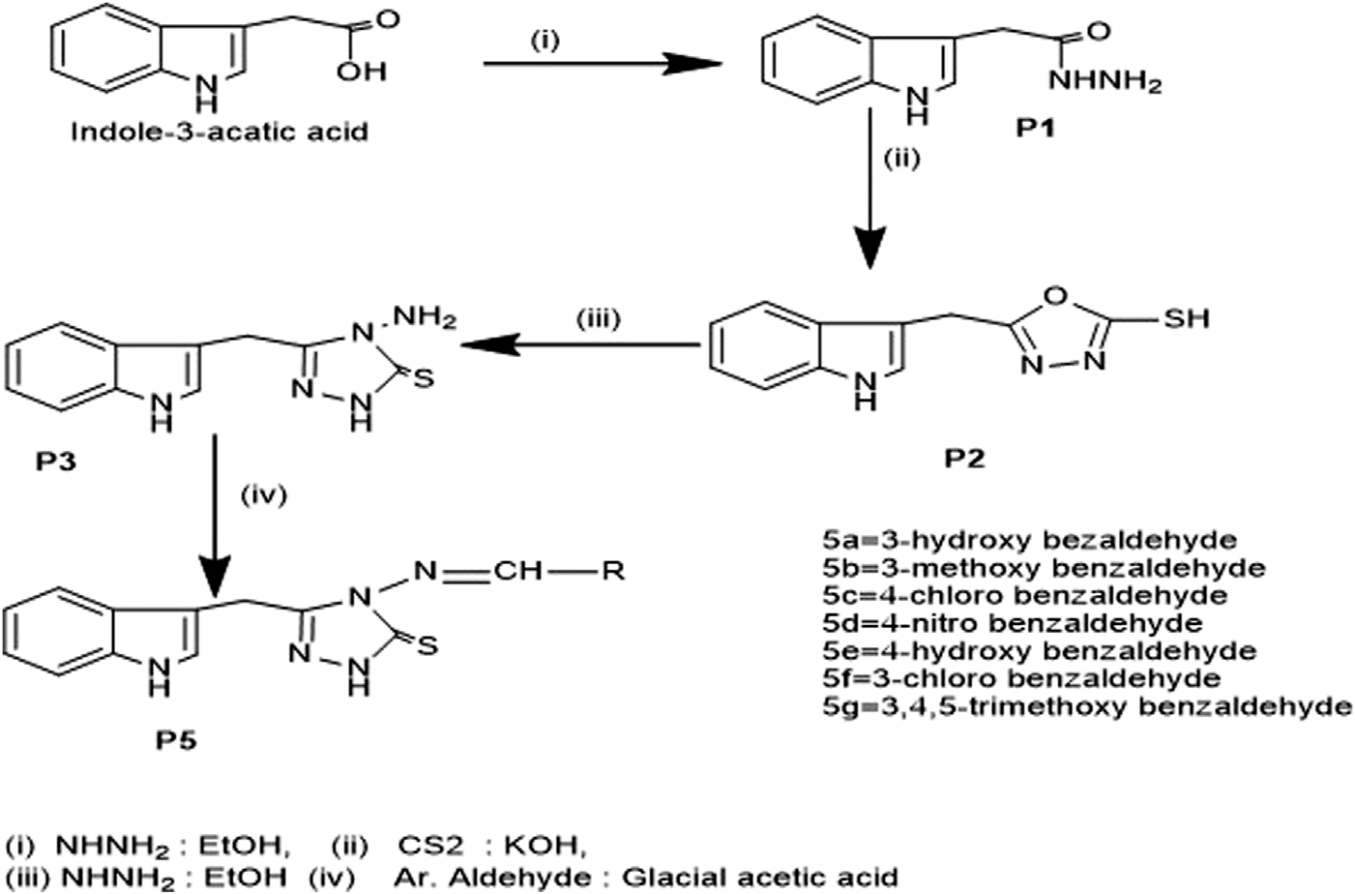
General Synthetic Scheme for synthesis of Schiff Bases (5a-5g).

**FIGURE 3:**
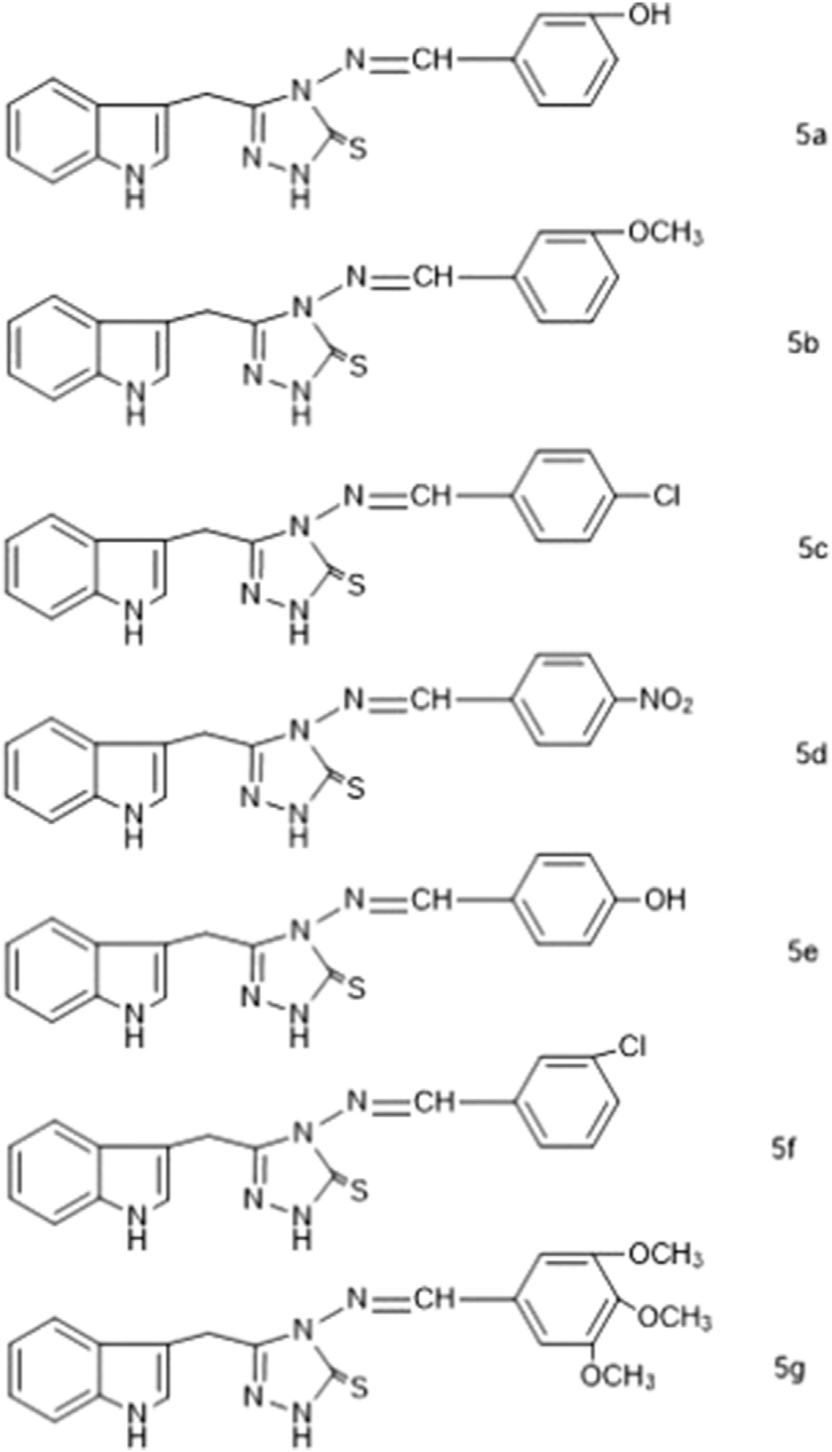
Proposed structures of synthesized compounds (5a-5g).

We synthesized the desired compounds according to scheme 1, following the method described by (Bayrak et al., 2009). The whole scheme is mentioned in Figure 2. Condensation of 1 with hydrazine in ethanol as reaction media yielded 2-(1H-indol-3-yl) acetohydrazide P1, In I.R., the C=O for acetohydrazide gives the impression at 1,620 cm^-1^, and for -NHNH_2_ around 3,260, 3,387 confirms the formation of P1. P2 was obtained from the reaction between P1 and CS_2_ in basic media; the final product was 1, 3, 4 -oxadiazole which was obtained by treatment of filtrate with conc. HCl and in FTIR were confirmed through characteristic peaks at 1,275 (-C=S) and 1,223 (-C-O-C) cm^-1^. P3; the key intermediate was the product of P2 and hydrazine hydrate reaction, confirmed *via* FTIR 1,277 (C=S), 1,629 (C=N) cm^-1^. P3 on treatment with aromatic_aldehydes in the ethanolic medium using Glacial acetic acid as catalyst yielded compounds 5a-5g. These Schiff base triazoles were confirmed through various spectroscopic techniques, i.e., FTIR, ^1^HNMR, and ^13^CNMR. The appearance of a singlet at 9.88–9.90 indicates -C=N for Schiff bases in ^1^HNMR, and at 158.30ppm for the same in ^13^CNMR, and at 1657cm^-1^ in FTIR, confirming it. During the synthesis of **5a-5g**, it was observed that substituted aromatic aldehydes possessing electron-withdrawing groups, e.g., -Cl, tend to end up reaction process early with good yield as compared to compounds affording electron-releasing groups, e.g., -OCH_3._ Figure 3 enlists the structures of synthesized compounds (**5a-5g**).

### 3.2 In silico studies

Prediction of compounds for their drug-like traits is critical to inquire about their pharmaceutical, pharmacokinetic and pharmacological behavior. Many drugs cannot go through clinical trials as per poor ADME (Adsorption, Distribution, Metabolism and Excretion) characteristics; in silico studies are acquiring an outstanding reputation in the drug development phase. (Arjun et al., 2019). Online tools were used to estimate the drug likeliness of newly synthesized compounds. SWISS ADME software was used to predict ADME properties; the data obtained are given in Table 1. All of these compounds are drug-like following RO5. In complete accordance with the Lipinski rule, any tested compound should fulfill the following mentioned conditions.

1. M.W. should be ≤500 Da.
2. HBA ≤10
3. HBD ≤5
4. iLOGP ≤5
5. MLOG ≤5

**TABLE 1:** Lipinski’s Rule of five for Drug Likeliness of compounds 5a-5g.

Compounds	MW	HBA	HBD	iLog po/w (iLOGP)	Log po/w (MLOGP)	P-gb substrate	CYP inhibition	Lipinski
								Violation
5a	349.41	3	3	2.27	1.98	No	CYP2C19, CYP2C9	None
5b	363.44	3	2	2.79	2.21	No	CYP1A2, CYP2C9, CYP2C19, CYP3A4	None
5c	367.86	2	2	2.85	3.03	No	CYP1A2, CYP2C9, CYP2C19, CYP3A4	None
5d	378.41	4	2	2.22	2.42	No	CYP2C19	None
5e	349.41	3	3	2.29	1.98	No	CYP2C9, CYP2C19	None
5f	367.86	2	2	2.81	3.03	No	CYP1A2, CYP2C9, CYP2C19, CYP3A4	None
5g	423.49	5	2	3.23	1.61	No	CYP2C9, CYP2C19, CYP3A4	None

MW, molecular weight; HBA, hydrogen bond acceptors; HBD, hydrogen bond donors; ilog Po/w, octanol-water partition coefficient; MLOGP, Moriguchi Log P (octanol-water partition co-efficient); P-gb, P-glycoprotein; CYP, Cytochrome P450.

**FIGURE 4:**
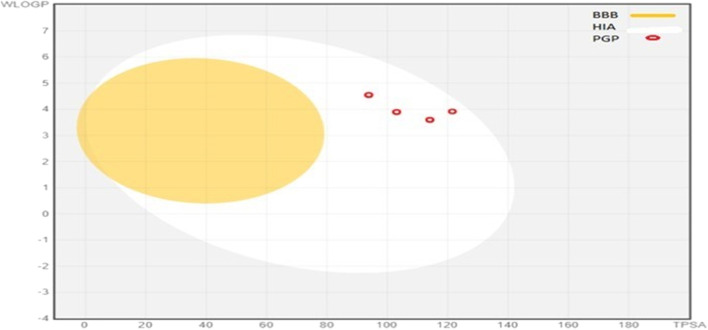
BOILED-Egg representations of absorption and distribution properties of (5a-5g) compounds. Egg white represen uman intestinal absorption (HIA), whereas egg yolk (yellow) shows blood-brain barrier permeability (BBB).

These given ADME parameters of compounds; help in assessing the potential of compounds for various factors, which may include distribution to the site of action, access to the bloodstream, and metabolization as non-toxic water-soluble excretes. SwissADME provides an insight into some basic parameters; solubility in water, ability to cross BBB, intestinal absorption, lipophilicity, attributes to bind with plasma proteins, and the ability to interfere with Cytochrome P 450 enzymes (Waseem et al., 2017). These compounds are prophesied for their CYP450 enzyme inhibition capacity, which accounts for CYP1A2, CYP2C19, CYP2C9, and CYP3A4. **5b, 5c,** and **5f** were able to inhibit all of these; **5g** inhibited 2C19, 2C9, and 3A4, **5a** and **5e** inhibited 2C19 and 2C9, **5d** inhibited 2C19 only. These findings state that these synthesized molecules may potentially affect the other drugs during metabolism, which are supposed to be metabolized by these enzymes. These compounds have a middling profile, can be investigated *in vivo*, and are selected for further inquiry. The BOILED-EGG method gives insight into the compound’s gut absorption and BBB permeability. In our synthetic library, all the compounds exhibited high gastrointestinal absorption. The appearance of a red dot indicates; being a non-substrate for globulin protein. The position of dots is linked with BBB permeability and gut absorption. The BOILED-Egg depiction of compounds is shown in Figure 4.

### 3.3 Pharmacologic evaluation

#### 3.3.1.Antioxidant assays

##### 3.3.1.1 DPPH assay

**FIGURE 5:**
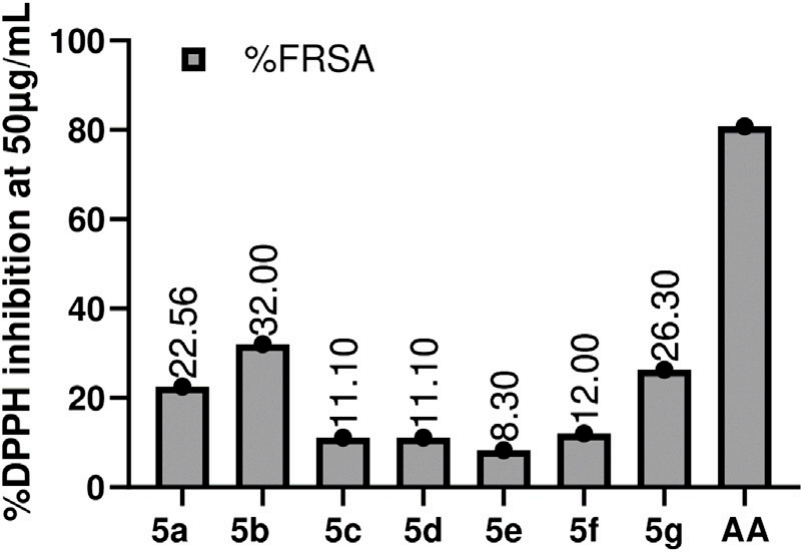
Graphical representation of DPPH Assay estimation of the synthesized compounds (5a-5g). Values given are expressed as the mean of triplicate ±standard deviation.

It is one of the very commonly utilized methods for the assessment of the antioxidant ability of compounds. DPPH turns from purple to yellow, which indicates the synthetic compound’s scavenging potential (Rahman et al., 2017). DMSO and A.A. were used as -ve and +ve control. The antioxidant potential of compounds was assessed at 50 μg/mL, 25 μg/mL, 12.5 μg/mL, and 6.25 μg/mL. All the compounds showed very slight antioxidant potential, with **5b (32%)** being the most active in the series, followed by **5a (22.55%),** then **5c** and **5d (11.1%)**, and the least active was **5e (8.3%).** The results are summarized in Figure 5. Production of free radicals is an outcome of regular oxygen consumption in the body; whenever something is missing with the natural antioxidant mechanism, the accumulation of these free radicals may lead to cytotoxic interaction with the body systems and may cause damage to genomic content of various cells, and proteins in the body (Barbuceanu et al., 2014). Free radical accumulation is also associated with different diseases, e.g., coronary heart disease, neuronic disorders, diabetes, necrosis, and various tumors (Ismaili.et al., 2008). The antioxidant potential of the compound depends upon its reducing power; the introduction of hydroxy and methoxy groups on the Benzene ring led to better reducing power or promising antioxidant activity (Rakesh et al., 2015). In this study, compounds **5b** and **5a** exhibited good antioxidant potential as having -OCH_3_ and groups at position three of the benzene ring. However, a change in position from three to four for Hydroxy benzaldehyde showed a significant loss in reducing potential. The basicity of the nitrogen atom also plays a significant role, i.e., the more basic is the nitrogen atom, the more active the compound will be in terms of its reducing potential (Hayun et al., 2018).

##### 3.3.1.2 Investigation of total antioxidant capacity (TAC) and total reducing power (TRP)

**FIGURE 6:**
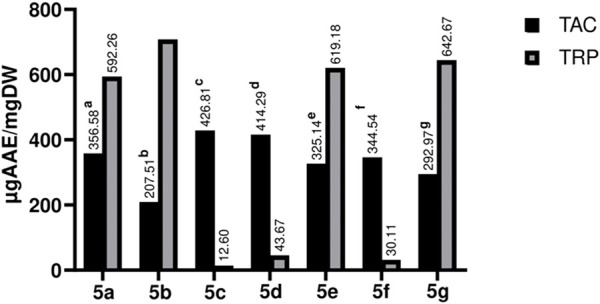
Graphical representation of TAC and TRP estimation of Library compounds (5a-5g). Values given are expressed as the mean of triplicate +standard deviation.

The antioxidant potential of test compounds was estimated by TAC &TRP assays. The spectrophotometric method was used to analyze the formation of the green-colored phosphomolibdenum compound. The newly synthesized compounds have shown noteworthy results in the TAC assay; **5b** showed the maximum activity among the synthetic compounds, whereas **5c** showed the least. The findings are depicted in Figure 6.

For estimation of reducing potential TRP assay was carried out, compound **5b** (706.42 ± 0.004 **µg AAE/mg**) showed the highest activity, followed by **5g** (642.67 ± 0.001 **µg AAE/mg**), **5e** (619.18 ± 0.010 **µg AAE/mg**), and **5a** (592.26 ± 0.007 **µg AAE/mg**). It is apparent from the results that compounds having hydroxy and methoxy in their structure showed promising activities as compared to those having nitro and chloro moieties at various positions. The primary mechanism of TRP assay is based on the compound’s capacity to reduce free radicals, which are generated during the procedure. i.e., Fe^3+^ is reduced to Fe^2+^ (Lee et al., 2015). Various factors determine the antioxidant potential of the compound; a few to explain include the chemical attributes, nature of antioxidant moieties, conditions and mechanism of the reaction, which accounts for the reason for a compound’s activity in one assay and none-to-less response in the other (Francenia Santos-Sanchez et al., 2019). However, compounds with hydroxyl and methoxy groups form the bases for potential candidates as an antioxidant (Rakesh et al., 2015).

#### 3.3.2 Anti-microbial screening

##### 3.3.2.1 Antibacterial assessment

Schiff base tri-azo compounds were investigated for antibacterial potential. The antibacterial assays were performed for nine different bacterial strains, which include; Gram-positive (*, S. aureus, B. subtilis*), Gram-negative (*Pseudomonas aeruginosa, K. pneumoniae, E. coli*)*,* and resistant bacterial strains (*S. aureus*, *MRSA*, *Pseudomonas aeruginosa, E. coli*. Compounds that exhibited significant activity, producing inhibitory growth zone ≥10 mm, their MIC values were determined (through broth microdilution method), ranging from 25 to 3.12 μg/mL. The Antibacterial activity of compounds and their minimum inhibitory concentration values are mentioned in Tables 2, 3. These results indicate that all the compounds showed *in-vitro* anti-microbial activity to some extent. None of the compounds in the given series was able to show inhibitory activity for *B. subtilis*, and only **5g** and **5f** were effective against *S. aureus* with MIC *25* μg/mL (**5a-5g**) showed activity against *K. pneumoniae*, and exhibited different values for MIC, ranging from 12.5 μg/mL for **5a, 5c, 5d**, and **5e**. 6.25 μg/mL for **5b** and **5g**, and the least, Table 4 3.12 μg/mL was for **5f**. **5a, 5b (MIC =**12.5 μg/mL)**, 5e** (MIC = 3.12 μg/mL, **same as Ciprofloxacin**) were active against *Pseudomonas aeruginosa,* and **5c, 5d, 5e** (MIC = 25 μg/mL) were active against E. *coli.* These compounds were effective against only one resistant bacterial strain, i.e., *MRSA*, and showed a MIC value of 12.5 μg/mL. Substituents with electron-withdrawing attributes, e.g., -NO_2,_ -Cl, -OH on the aromatic ring, assert a great impact on the antibacterial activity of synthetic compounds. It is also observed that an increase in the number of -OH groups tend to enhance the activity (Hosamani and Shingalapur, 2011). Due to the difference in cell-wall composition of Gram + ve and -ve bacteria, different compounds show different features towards the same micro-organism (Goszczyn’ska et al., 2015).

**TABLE 2:** Antibacterial evaluation of the synthetic compounds of Library compounds (5a-5g) against gram-positive and gram-negative bacteria and their MIC values.

Compounds	Antibacterial activity (25 μg/mL)										
	Gram + ve				Gram-ve						
	*S. aureus*		*Bacillus subtilis*			*Pseudomonas aeruginosa*		*Klebsiella pneumoniae*		*E. coli*	
	Activity	MIC μg/mL	Activity	MIC μg/mL		Activity	MIC μg/mL	Activity	MIC μg/mL	Activity	MIC μg/mL
5a	-----	N.A.	-----	NA		Active	12.5	Active	12.5	_	NA
5b	---	N.A.	---	NA		Active	12.5	Active	6.25	---	NA
5c	---	N.A.		N.A.		---	NA	Active	12.5	Active	25
5d	---	N.A.		N.A.		---	NA	Active	12.5	Active	25
5e	---	N.A.	---	NA		Active	3.12	Active	12.5	Active	25
5f	Active	25	---	N.A.		---	NA	Active	3.125	---	NA
5g	Active	25	---	N.A.		---	NA	Active	6.25	---	NA
Ciprofloxacin	Active	3.12	Active	3.12		Active	3.12	Active	3.12	Active	3.12
DMSO	---	NA	---	NA		---	NA	---	NA	---	NA

Values are mean ± SD, *n* = 3; ≥ 10 mm zone of inhibition was considered for MIC. Positive control = Ciprofloxacin; Negative control = DMSO; Active = Bacterial activity; --- = no activity, NA=not applicable, (−) indicates not applied.

**TABLE 3:** Antibacterial evaluation of the synthetic compounds of Library compounds (5a-5g) against resistant gram-positive and gram-negative bacteria and their MIC values.

Compounds	Antibacterial activity (25 μg/mL)							
	Gram + ve				Gram -ve			
	*S. aureus*		MRSA		*P. aeruginosa*		E.coli	
	Activity	MIC μg/mL	Activity	MIC μg/mL	Activity	MIC μg/mL	Activity	MIC μg/mL
5a	-----	NA	Active	12.5	-----	NA	-----	NA
5b	---	NA	Active	12.5	---	NA	---	NA
5c	---	NA	Active	12.5	---	NA	---	NA
5d	---	NA	Active	12.5	---	NA	---	NA
5e	---	NA	Active	12.5	---	NA	---	NA
5f	---	NA	Active	12.5	---	NA	---	NA
5g	---	NA	Active	12.5	---	NA	---	NA
Ciprofloxacin	Active	3.12	Active	3.12	Active	3.12	Active	3.12
DMSO	---	NA	---	NA	---	NA	---	NA

Values are mean ± SD, *n* = 3; ≥ 10 mm zone of inhibition was considered for MIC, Positive = have anti-bacterial activity; --- = no anti-bacterial activity; Positive control = Ciprofloxacin (10 μg/mL). Negative control, DMSO.

**TABLE 4:** Antifungal evaluation of the synthetic compounds (5a-5g).

Serial no	Compounds	Zone of inhibition (mm)				
		*Mucor*	*A. niger*	*A. flavus*	*A. fumigatus*	*F. solani*
1	5a	---	---	---	---	---
2	5b	---	---	---	---	---
3	5c	---	---	---	---	---
4	5d	---	---	---	---	---
5	5e	---	---	---	---	6
6	5f	---	---	---	---	---
7	5g	---	---	---	---	---
8	Amphotericin B	11.5 mm	12.5 mm	12 mm	10 mm	11.5 mm
9	DMSO	---	---	---	---	---

Values are mean ± SD, *n* = 3; sample concentration= 20 μg/disc, --- = no anti-fungal activity; positive control concentration = 20 μg/disc, Positive control = Amphotericin B, Negative control = DMSO.

##### 3.3.2.2 Antifungal estimation

The Synthetic compound library was subjected to an antifungal assay using five different strains of Fungai, i.e., *Fusarium solani* (FCBP-0291), *Aspergillus fumigatus* (FCBP-66), *Aspergillus flavus* (FCBP-0064), *Aspergillus niger* (FCBP-0198) and Mucor species (FCBP-0300). Amphotericin B was the standard drug used. None of the compounds showed antifungal activity, except **5e**, which was active against only one strain of *Fusarium solani*. The outcomes are concise in Table 5.

**TABLE 5:** Protein Kinase inhibitory potential of compounds (5a-5g).

Serial no	Compounds	Bald zone (mm)	Clear zone (mm)
1	5a	9	6
2	5b	10.5	7.5
3	5c	---	---
4	5d	---	---
5	5e	---	---
6	5f	---	---
7	5 g	7.5	6
8	Surfactin B	22.3	---
9	DMSO	---	---

Surfactin B (20 μg/mL) = positive control; --- = no activity; DMSO, negative control; Sample Concentration = 20 μg/mL.

### 3.3.3.Cytotoxic evaluation

#### 3.3.3.1.Brine shrimp lethality assay

**FIGURE 7:**
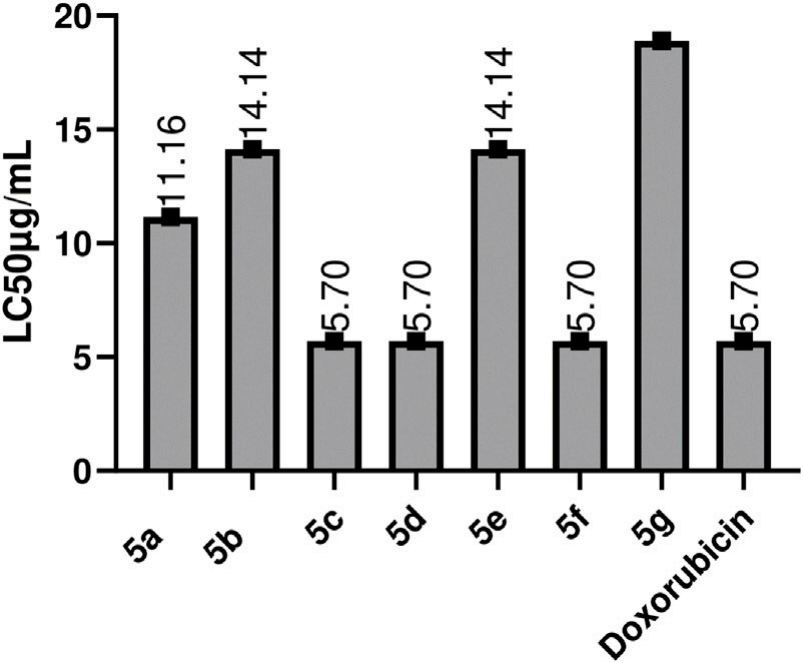
Toxicity evaluation of the synthetic compounds by using Brine shoxorubicin Values are expressed as Mean ± SEM(standard error of the mean, *n* = 3) lethality assay.

This evaluation test forms the basis for the bioactive scanning of compounds for their potential as anti-cancer moieties, to be investigated on a large scale (Amanullah et al., 2012). Through research, it has been found that the compound’s anti-cancer potential and B.S. lethality has direct relation (El-Gohary and Shaaban, 2014). Similarities between *Artemia salina* and mammalian cells have been reported due to DNA-dependent RNA polymerases (Birndorf et al., 1975). These Schiff bases were investigated for their cytotoxic potential. The values for the toxicity test of all 1, 2.4-Triazole compounds against brine shrimp assay and LC_50_ values are described in Figure 7. Doxorubicin was used as the positive control. All of the compounds showed cytotoxic potential, and a few expressed activities similar to standard; doxorubicin, some have less value of LC_50_ than Doxorubicin **5c, 5d, and 5f** showed max. Activity with LC_50_ values of 5.7 μg/mL, followed by **5b, 5e** 14.14 μg/mL, and the maximum value was observed for **5g**, which is 18.18 μg/mL. Compounds that possess -Cl, -OH, at 2, 3, and -NO_2_ at position four at the side chain of the main nucleus show good cytotoxic potential (Hosamani and Shingalapur, 2011). The substantial LC_50_ values of Schiff bases indicate that these possess cytotoxic capacity, which allows further studies to be carried -out. From a pharmacologic point of view, it is pertinent that the compounds with promising activity in B.S. assay are well-thought-out as better antitumoral agents (Carballo et al., 2002).

##### 3.3.3.2 Protein kinase inhibition assay

These synthetic products were evaluated to estimate the inhibitory potential for protein kinase of Streptomycin 85 E. Protein kinase is involved with the mycelium production of this species, so the growth patterns were observed as the bald zone. The clear zone specifies no cytotoxic bacterial growth; as far as the bald zone is concerned, it shows inhibition of protein kinase. The results for this assay for compounds (**5a-5g**) are given in Table 5. One of the propitious targets in cancer treatment is the inhibition of protein kinases. Protein phosphorylation by protein kinases at various residues regulates diverse biological processes, which may include apoptosis, proliferation and differentiation of cells. Deregulation at any step in early tumorigenesis may lead to cancer (Yao et al., 2011). Aerial hyphae formation in *Streptomyces* depends upon protein kinase activity; this scenario is exploited in this test; to inquire about these compounds’ kinase inhibition potential and to look at their anti-cancer potential. The advantage of using *Streptomyces* for kinase inhibition assay is its predisposition to a wide range of eukaryotic cells. Whole-cell assay of *Streptomyces* not only identifies the compound’s cytotoxic potential but also identifies signal transduction inhibitors for various activities, e.g., antitumor, anti-mycobacterial, and and anti-infective (Waters et al., 2002). Among the tested compounds, only **5a, 5b,** and **5g** showed inhibitory activity. **5b** showed the max. Activity with a bald zone of 10.5 mm and a clear zone of 7.5 mm, following is 5a with a bald zone of 9 mm and a clear zone of 6 mm, and for 5g, values are 7.5 and 6 mm, respectively. It means that these compounds have the potential for protein kinase inhibition as well as the toxicity of the compound.

## 4 Conclusion

The synthesized Schiff bases showed encouraging results in these assays. The whole series was found to follow Lipinski’s rule of five. Among these synthesized compounds, no one was able to show promising activity in DPPH assay; **5b** showed max. Antioxidant potential in TAC and TRP assays. In antibacterial evaluation, **5e** was effective against max. the number of the strains, which include *P. aeruginosa*, *K. pneumoniae*, *E. coli*, *MRSA*, **5g,** and **5f,** were active for Gram + ve, Gram -ve, and resistant strains, with MIC values comparable to reference drug Ciprofloxacin. **5b** was most active in series as a Protein kinase inhibitor, and **5c, 5d,** and **5f** showed max. Cytotoxic potential in Brine shrimp lethality assay with LC_50_ 5.7 μg/mL, equal to Doxorubicin. SAR studies reveal that the -OH group plays a vital role. Compounds with -Cl, -NO_2,_ and -OH are good candidates for cytotoxic potential. In short, this study has revealed the scope for further research in these areas to develop novel bioactive compounds and launch rational QSAR studies.

## Data Availability

The original contributions presented in the study are included in the article/supplementary material, further inquiries can be directed to the corresponding author.
